# The Relationship Between Exacerbation of Pachymeningitis and Chemotherapy: A Case Report

**DOI:** 10.7759/cureus.25792

**Published:** 2022-06-09

**Authors:** Maisha Maliha, Zinath Roksana, Shimul A Babli, Khaza Chowdhury, David West

**Affiliations:** 1 Internal Medicine, Dhaka Medical College, Dhaka, BGD; 2 Internal Medicine, Feni Sadar Hospital, Feni, BGD; 3 Internal Medicine, Islami Bank Medical College and Hospital, Rajshahi, BGD; 4 Internal Medicine, Lee Health, Florida, USA

**Keywords:** inflammation, cytokine storm, exacerbation, chemotherapy, pachymeningitis

## Abstract

Pachymeningitis is a rare disorder that involves the dura mater of the cranial and spinal nerves. It can lead to localized or diffuse thickening of the dura mater as an inflammatory reaction. Very little is known about this uncommon disease, and even less is known about its exacerbating factors and relationship with chemotherapy.

In this report, we present a case of an 86-year-old man with metastatic bladder carcinoma on chemotherapy who experienced worsening pachymeningitis with symptoms such as headache, aphasia, weakness, and seizures. The patient responded well to steroids, and his symptoms improved. This association between exacerbation of pachymeningitis and chemotherapy is rarely encountered, and its mechanism of action is poorly understood. We hope this case report will add to the existing literature on this uncommon phenomenon and its exacerbating factors.

## Introduction

Pachymeningitis is an unconventional disease that can cause diffuse or localized thickening of the dura mater [1]. It can be broadly classified into two different types: ‘primary’ or ‘idiopathic hypertrophic pachymeningitis,’ where no causes of the disease are identified, and ‘secondary’ in which it is associated with other systemic illnesses [1]. The most commonly associated conditions are infectious diseases such as tuberculosis, syphilis, Lyme disease, and inflammatory conditions such as sarcoidosis, IgG4-related disease, and Wegener’s granulomatosis [2]. It is also rarely associated with neurosarcoidosis and certain cancers [3].

The commonly encountered symptoms of pachymeningitis include headache, cranial nerve palsy, visual problems, cerebellar ataxia, and facial pain [3]. Other rare manifestations include hearing disorders and audiovestibular abnormalities [4]. The various steps in diagnosing the disease include imaging, especially MRI, cerebrospinal fluid analysis, rheumatological tests, immunology, and meningeal biopsy [5]. The confirmation of diagnosis involves MRI and meningeal biopsy [5]. The primary modalities of treatment are glucocorticoids and immunosuppressive therapy [5].

There is very little information about the exacerbating factors of pachymeningitis and its relationship with chemotherapy. To our knowledge, this is the first report demonstrating a significant worsening of pachymeningitis in a patient while on chemotherapy.

## Case presentation

**Figure 1:**
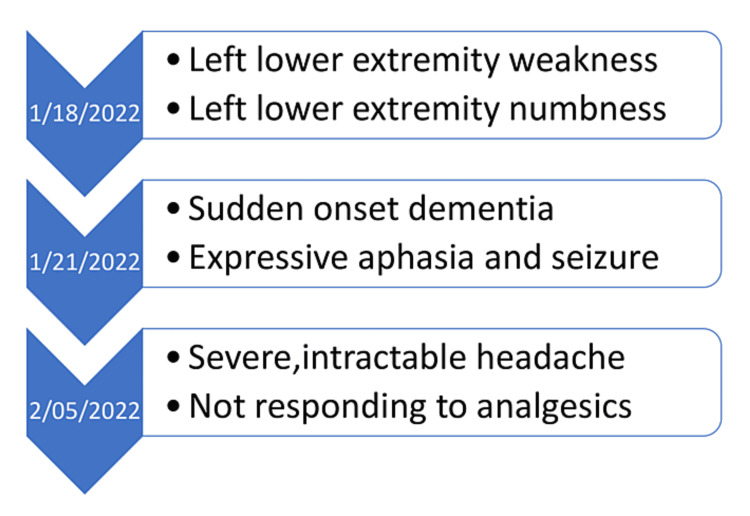
Figure showing sequence of events in the patient

An 86-year-old Caucasian male presented to our hospital with metastatic bladder cancer for treatment with chemotherapy. The chemotherapy regimen consisted of gemcitabine 1000 mg/m^2 ^IV on days one, eight, and 15, cisplatin 70 mg/m^2^ IV on days one and two, followed by maintenance therapy of avelumab 800 mg IV. The cycle was repeated every two weeks. The patient initially responded well to the treatment. He had a history of hypertension, hyperlipidemia, hypothyroidism, right lower extremity deep vein thrombosis, and coronary artery disease. He had been previously diagnosed with asymptomatic idiopathic hypertrophic pachymeningitis four years back. During a year of receiving chemotherapy, the patient experienced progressive left lower extremity weakness and numbness. It was followed by the development of sudden onset of dementia, expressive aphasia, and absence seizure. Afterward, the patient developed a severe, intractable headache that was not responsive to analgesics. The sequence of events is shown in Figure 1.

**Figure 2:**
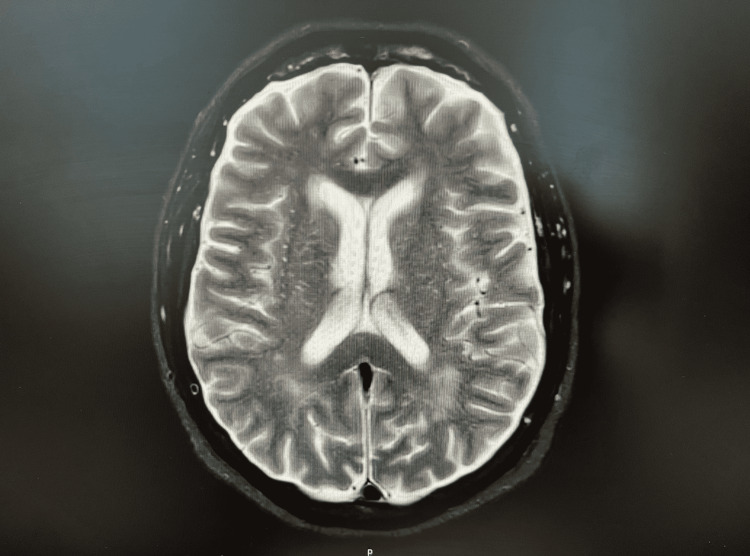
MRI of head showing dural enhancement MRI: magnetic resonance imaging

Imaging such as MRI showed worsening pachymeningitis; there was a significant increase in dural thickening and enhancement, extending inferiorly into the cervical cord region and odontoid process, as seen in Figure 2.

There was also substantial involvement of the pons, cerebellopontine angle, and cisterns. There was no evidence of hemorrhage or new infarcts on the MRI or CT scan. In addition, the cerebrospinal fluid analysis showed an elevated protein level and an increase in the level of oligoclonal bands. IgG4, ACE, ANA, rheumatoid factor, erythrocyte sedimentation rate, and C-reactive protein levels were within normal limits. Infectious diseases such as syphilis, tuberculosis, and Lyme disease were ruled out by performing venereal disease research laboratory (VDRL) test, interferon-gamma release assay (IGRA), and chest X-ray respectively, and ELISA for Borrelia burgdorferi.

**Figure 3:**
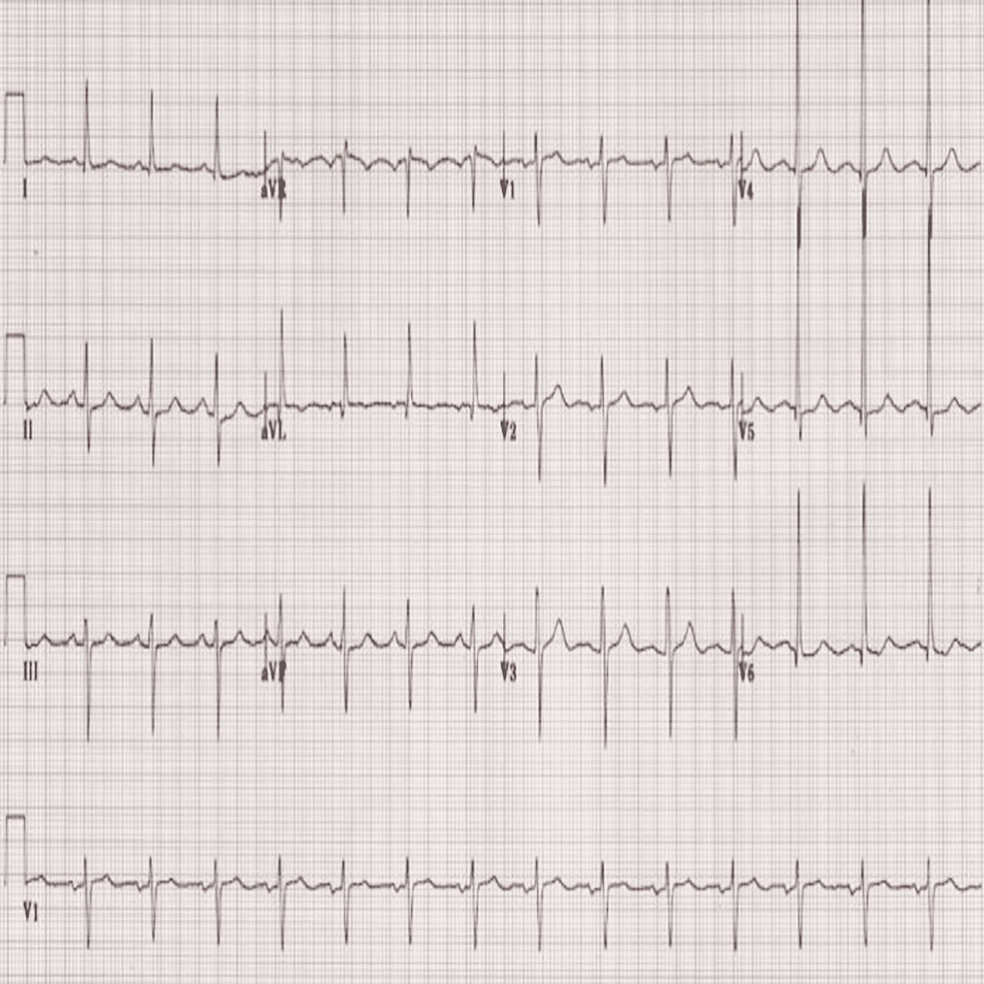
ECG showing left ventricular hypertrophy ECG: electrocardiogram

Among other investigations, the patient's abdominal ultrasonography showed his extensive bladder carcinoma. His ECG showed left ventricular hypertrophy, and ST-segment depression features consistent with his history of prolonged hypertension and coronary artery disease, as shown in Figure 3.

His lipid profile showed slightly elevated levels of cholesterol and low-density lipoprotein. Other routine investigations revealed no significant changes.

The patient was started on intravenous methylprednisolone 1 gm and 100 mg oral prednisone and was slowly tapered till his symptoms improved. His headache gradually improved significantly, and his weakness and numbness resolved completely. However, his dementia and aphasia persisted despite the treatment. His other medications included aspirin, levothyroxine, enoxaparin, apixaban, and atorvastatin.

## Discussion

Pachymeningitis is a sporadic disease [6], and its incidence and prevalence are very low in the US population [6]. Very few cases have been reported about pachymeningitis and its exacerbating factors in the literature. The relationship between chemotherapy and the worsening of pachymeningitis is a rare phenomenon that can cause significant impairment.

Pachymeningitis is primarily an amalgamation of inflammatory and fibrosing conditions [7]. The process includes the activation of the immunological pathway consisting mainly of B and T lymphocytes [7]. This leads to activation of fibroblasts that results in collagen deposition and hypertrophy of tissues, and ultimately leads to the thickening of the dura and neurological impairment [8]. The classical pathological findings include lymphoplasmacytic infiltration, spindle-shaped fibrosis, and obliterative phlebitis [8]. Since it is an inflammatory condition, the treatment modalities include immunosuppressive and anti-inflammatory drugs such as corticosteroids and rituximab [7].

Cancer chemotherapy provides effective curative treatment for various cancers [9]. Cisplatin-based chemotherapy has been shown to be highly effective in treating metastatic bladder cancer [10]. Evidence from animal studies and clinical cohorts has shown that cancers are stimulated to release pro-inflammatory cytokines, chemokines, and other inflammatory mediators in response to chemotherapy [11]. This has been described as a ‘cytokine storm’ that helps cancer cells evade apoptosis and aid in further invasion and metastasis when released into the peripheral circulation [12]. The most commonly released inflammatory mediators include tumor necrosis factor-alpha (TNF-α), C-C motif chemokine ligand-2 (CCL2), and interleukin 6 (IL-6) [11].

Since pachymeningitis is an inflammatory condition, it can be theorized that the pro-inflammatory state created in response to chemotherapy resulted in the aggravation of pachymeningitis and the progression of the disease in our case. Hence, the cytokine storm created by chemotherapy was the prime reason for the worsening of pachymeningitis and led to the patient’s profound neurological impairment. Since the cytokines were released in increasing progression, the patient developed rapid worsening of symptoms over a year that caused him significant distress. 

Steroids have a significant anti-inflammatory effect [13]. Their primary mode of action includes regression of transcription of genes encoding pro-inflammatory cytokines and chemokines [13]. Hence, it can be theorized that steroids suppressed the effect of the cytokine storm and thus helped improve the symptoms of the patient. This further supports the hypothesis that the cytokine storm generated by chemotherapy was the primary reason behind the worsening of pachymeningitis.

## Conclusions

Pachymeningitis can be markedly aggravated by the inflammatory mediators produced by cancer cells in response to chemotherapy. More research is required to explore this relationship, which will help in ensuring early diagnosis and better quality of life for the patients. We hope that this case report will guide physicians to understand the interaction between chemotherapy and pachymeningitis. This will help them to be more prudent when choosing the cancer treatment modalities in the setting of pachymeningitis.
